# The Public Perception of the #GeneEditedBabies Event Across Multiple Social Media Platforms: Observational Study

**DOI:** 10.2196/31687

**Published:** 2022-03-11

**Authors:** Congning Ni, Zhiyu Wan, Chao Yan, Yongtai Liu, Ellen Wright Clayton, Bradley Malin, Zhijun Yin

**Affiliations:** 1 Department of Computer Science Vanderbilt University Nashville, TN United States; 2 Department of Biomedical Informatics Vanderbilt University Medical Center Nashville, TN United States; 3 Center for Genetic Privacy & Identity in Community Settings Vanderbilt University Medical Center Nashville, TN United States; 4 Center for Biomedical Ethics and Society Vanderbilt University Medical Center Nashville, TN United States; 5 Department of Pediatrics Vanderbilt University Medical Center Nashville, TN United States; 6 School of Law Vanderbilt University Nashville, TN United States; 7 Department of Biostatistics Vanderbilt University Medical Center Nashville, TN United States

**Keywords:** CRISPR/Cas9, gene-edited babies, social media, stance learning, text mining, content analysis

## Abstract

**Background:**

In November 2018, a Chinese researcher reported that his team had applied clustered regularly interspaced palindromic repeats or associated protein 9 to delete the gene *C-C chemokine receptor type 5* from embryos and claimed that the 2 newborns would have lifetime immunity from HIV infection, an event referred to as #GeneEditedBabies on social media platforms. Although this event stirred a worldwide debate on ethical and legal issues regarding clinical trials with embryonic gene sequences, the focus has mainly been on academics and professionals. However, how the public, especially stratified by geographic region and culture, reacted to these issues is not yet well-understood.

**Objective:**

The aim of this study is to examine web-based posts about the #GeneEditedBabies event and characterize and compare the public’s stance across social media platforms with different user bases.

**Methods:**

We used a set of relevant keywords to search for web-based posts in 4 worldwide or regional mainstream social media platforms: Sina Weibo (China), Twitter, Reddit, and YouTube. We applied structural topic modeling to analyze the main discussed topics and their temporal trends. On the basis of the topics we found, we designed an annotation codebook to label 2000 randomly sampled posts from each platform on whether a supporting, opposing, or neutral stance toward this event was expressed and what the major considerations of those posts were if a stance was described. The annotated data were used to compare stances and the language used across the 4 web-based platforms.

**Results:**

We collected >220,000 posts published by approximately 130,000 users regarding the #GeneEditedBabies event. Our results indicated that users discussed a wide range of topics, some of which had clear temporal trends. Our results further showed that although almost all experts opposed this event, many web-based posts supported this event. In particular, Twitter exhibited the largest number of posts in opposition (701/816, 85.9%), followed by Sina Weibo (968/1140, 84.91%), Reddit (550/898, 61.2%), and YouTube (567/1078, 52.6%). The primary opposing reason was rooted in ethical concerns, whereas the primary supporting reason was based on the expectation that such technology could prevent the occurrence of diseases in the future. Posts from these 4 platforms had different language uses and patterns when they expressed stances on the #GeneEditedBabies event.

**Conclusions:**

This research provides evidence that posts on web-based platforms can offer insights into the public’s stance on gene editing techniques. However, these stances vary across web-based platforms and often differ from those raised by academics and policy makers.

## Introduction

### Background

The clustered regularly interspaced palindromic repeats (CRISPR) or CRISPR-associated protein 9 (Cas9) genetic scissor is a revolutionary gene-editing technology for the life sciences, for which its creators were awarded the 2020 Nobel Prize in Chemistry [1]. However, much remains unknown about the long-term effects of applying CRISPR in *human gene editing* (HGE), and experts in ethics and policy have particularly cautioned against its use in *germline gene editing* (GGE) to influence the genetic code of a human embryo [2,3]. However, in November 2018, a Chinese researcher, Jiankui He, reported that his team applied this tool to delete the *gene C-C chemokine receptor type 5* from embryos and claimed that the 2 newborns would benefit from lifelong immunity from HIV infection. This event, which is often referenced as #GeneEditedBabies in the web-based domain, has raised widespread concerns in the scientific community about HGE, especially GGE, for numerous reasons, which include but are not limited to biological safety and ethical implications [4-6].

The experiments of Jiankui He violated Chinese regulations and ignored international ethical norms [7]. Most criticisms against his actions concentrated on assertions that the experiments (1) were unnecessary as existing treatment strategies can sufficiently block HIV paternal transmission without the risk of GGE [8] and (2) violated the protocols required for general genetic procedures [9], expressing a concern that off-target editing can lead to dangerous and unpredictable mutations [10,11]. To expedite global cooperation on science and its governance, an international commission comprising experts from 10 countries released a guidance report in September 2020 on how to determine whether such an application is sufficiently well-developed for clinical use [12].

However, the worldwide debate has focused primarily on the perspective of academics and policy makers. By contrast, little is known about how the public, who are often influenced by geographic region and local culture, perceived this event, as well as these issues more generally. Gaining intuition into the public’s perspectives on these matters is critical to understanding social norms and expectations for potential beneficiaries and victims. In this respect, the public’s perspective is a core component in determining resource allocation, political policies, and participation in clinical trials, all of which influence the development and adoption of such technologies as they evolve [13,14].

To date, research on public attitudes has mainly applied survey methods to investigate hypothetical personal opinions regarding gene-editing techniques [15-18], and few have considered this specific actual event. At the same time, web-based social media platforms have become an essential medium for billions of people to learn about and comment on trending events, including #GeneEditedBabies, in a timely manner, making them an ideal resource to study public perception. Web-based social media platforms accommodate speech from wide demographics, and the discussions and expressions therein are not controlled by predefined questionnaires. Thus, they may effectively reduce the bias in the data [19]. As a result, over the past decade, user-generated content from web-based social media platforms has been increasingly relied upon to study the public’s attitudes regarding a broad range of topics, including weight stigmatization [20], antivaccination [21], lung cancer screening [22], the Brexit referendum [23], and political debates [24]. A total of 3 recent studies of web-based data investigated the #GeneEditedBabies event; however, they were limited in that they either focused only on sentiment [25] or relied on data from only 1 web-based platform [26,27], which, as we show in this study, paints an incomplete and likely biased picture.

### Objective

In this work, we present a substantially broader investigation into the public’s perceptions of the #GeneEditedBabies event through the lens of four popular social media platforms known to be influenced by different cultures and demographics: *Sina Weibo* (based in China), *Twitter*, *Reddit*, and *YouTube*. Specifically, we apply topic modeling to >220,000 posts to analyze what had been discussed about this event across these platforms and examine how these topics changed over time. Our results indicate that the main topics were related to news; the technique itself; posting words on the web; and discussions from the perspectives of disease, risk, laws, ethics, and scientific literature. We also observed that certain topics have clear temporal trends based on the heat of the relative discussions. On the basis of the topic analysis results, we further design a codebook to annotate 2000 randomly selected posts from each platform, which we use to investigate how web-based posts supported, opposed or held a neutral stance on this event. The results indicate that the public’s web-based posts had much more divided stances toward this event than toward those of academics, although the former varied across platforms with different language patterns.

The findings of this study indicate that society can learn about the public perception of controversial events using web-based data; however, we must be careful about drawing conclusions from any single platform.

## Methods

### Overview

Our research focused on the data that were collected from four popular and publicly accessible web-based platforms with user bases from different regions and backgrounds: Sina Weibo, Twitter, YouTube, and Reddit. The internal review board at our university designated this project as non–human subjects research and exempt from review. All posts presented in this paper have been rephrased for privacy concerns and demonstration purposes.

### Data Preparation

As the #GeneEditedBabies event was instigated by a Chinese scientist, it is natural to examine how people reacted to this event on Sina Weibo, the most popular microblogging platform with 500 million users in China [28]. The data from this platform have been applied to investigate a broad range of topics, including suicide prediction [29] and the mining of characteristics of patients with COVID-19 in China [30].

Twitter and YouTube are web-based platforms with a broader international basis, where approximately 20% of their users are from the United States, and the other 80% are from other countries [31]. Owing to its instant posting feature, Twitter has been recognized as an ideal platform for tracking people’s comments or attitudes toward social events over time [32,33]. YouTube, a web-based video-sharing platform that enables users to upload, view, rate, and comment on videos has also become a valuable resource for studying the public’s stances [34,35].

Reddit is a social news aggregation, web content rating, and discussion website, with approximately 50% of users from the United States [36]. Reddit users are more likely to write longer posts and generate deeper discussions on specific topics than users from other platforms. In addition, most Reddit users reportedly have a college degree [37].

We acknowledge that the users who mentioned the #GeneEditedBabies event do not necessarily represent the general population in each platform and, thus, may not represent the same demographics as those previously mentioned. However, because of the wide coverage of their user base, we believe that the posts on these platforms can provide intuition into the public’s stance regarding the #GeneEditedBabies event in a cross-regional and cross-cultural manner [38].

To collect data, we defined a set of keywords that were related to the #GeneEditedBabies event: *jiankui he*, *gene-edited babies*, *crisper babies*, *贺建奎*, *基因编辑婴儿*, and their variations. Given that each platform has distinct application programming interface (API) use policies and that we initiated data collection 3 days after the event (November 29, 2018), we relied upon several different strategies to collect data. Specifically, we used the Twitter streaming API to continuously collect data from the Twitter stream and used the Twitter search API to fetch the data that were generated before the initial data collection date. For the other web-based platforms, we used their search APIs to collect data. We collected data from November 26, 2018, to October 19, 2019; aggregated the data on each platform; and removed duplicates. For Twitter, YouTube, and Reddit, we focused only on posts written in English.

### Topic Analysis

To characterize what has been discussed in the #GeneEditedBabies event and provide a basis for the following stance annotation, we conducted a topic analysis of all collected data using structural topic modeling (STM) [39]. STM is a topic modeling framework that can leverage document-level meta-information (eg, publication date and author) to improve inference and qualitative interpretability. However, because of language differences, it is challenging to generate topics by applying a single model to all the data. In addition, as many posts and comments in YouTube data do not have a well-defined posting date (eg, *one month ago*), we could not accurately build temporal trends for this platform’s topics. As a result, we applied STM on Twitter and Reddit data sets together to (1) extract general topics, (2) analyze topic trends, and (3) compare topic differences; we applied STM on Sina Weibo and YouTube data sets to extract only general topics separately. We believe that temporal analysis can help understand how related news triggered the public’s discussions on web-based social media platforms.

We removed stop words, punctuation, and numbers and replaced words with their stems before applying STM. As STM is based on unsupervised learning, we relied on exclusivity and semantic coherence to select the optimal number of topics K^*^ by running STM, with each candidate for the number of topics ranging from 5 to 25. Exclusivity corresponds to the distinctiveness of words with the highest probabilities in the topics, whereas semantic coherence measures the probability of co-occurrence of these words in a topic. As large exclusivity often results in small semantic coherence and vice versa, an ideal choice of K^*^ should strike a balance between these 2 measures. To do so, we first selected the 3 topic number candidates for K^*^ that exhibited the largest proportion of topics, with scores within 1 SD of the mean for the metrics of interest. The mean score was calculated for all the candidate topic numbers. We then manually compared and selected the number of topics that resulted in the most interpretable topics through the authors’ observations and discussions.

### Stance Annotation

#### Overview

Topic analysis can provide a high-level picture of what has been discussed in general. However, it cannot precisely tell whether people support or oppose this event. Therefore, after topic analysis, we conducted a stance analysis to further investigate how web-based posts from different platforms reacted to this event.

#### Coding Question

To identify and analyze the stance of web-based users in the #GeneEditedBabies event, we randomly selected 2000 posts from each platform for annotation. We designed a codebook for data annotation based on reading, independently summarizing, and collectively discussing the topic analysis results among the authors (CN, ZW, CY, and ZY) of this manuscript. We formulated 2 annotation tasks for each selected post. First (question 1 [Q1]), we asked what the post’s stance was toward this event and let annotators select one of the following options: *no clear stance*, *support*, *oppose*, or *neutral*. As the distinction between *no clear stance* and *neutral* stances can be somewhat nuanced, we instructed annotators that a post with *a neutral* stance must explicitly balance between supporting and opposing stances.

If the answer to Q1 was different from *no clear stance*, we then asked annotators to perform a second task (question 2 [Q2]) to identify what issues the post raised when expressing its stance. Q2 was a nonexclusionary multi-option question, in which annotators were instructed to choose any of the following options based on the instructions of the codebook: (1) *techniques* (eg, considering the development of science or technologies), (2) *ethics*, (3) *laws*, (4) *judging Jiankui He* (eg, on his education, appearance, or ambition), (5) *judging the organization* (eg, He’s institute or country), (6) *others* (eg, those not listed above, but the missing aspect should be recorded), and (7) *no reasons are mentioned*. Table 1 presents several examples along with their answers.

**Table 1:** Examples of posts with answers to the questions posed in the annotation tasks.

Post examples	Platform	Q1^a^ choice (suggested)	Q2^b^ choice (suggested)
“年轻有为， 反正我不会这个技术。 (He is young and promising; I do not understand this technology anyway)”	Sina Weibo	Support	Techniques and judging Jiankui He
“The first gene-edited babies claimed in China—The Mainichi https://t.co/uNL0QFfdur”	Twitter	No clear stance	N/A^c^
“I mean Nazi scientists went to hell in a tote basket but produced some of the most influential research of the 20th century.”	Reddit	Neutral	Techniques and ethics
“It sounds like any other mad scientist story.”	YouTube	Oppose	Judging Jiankui He

^a^Q1: question 1.

^b^Q2: question 2.

^c^N/A: not applicable.

#### Annotation Strategies

**Figure 1:**
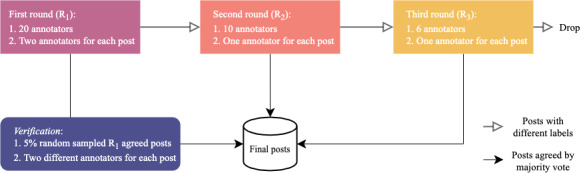
Pipeline of the 3-round annotation and verification process.

As the languages used in the 4 platforms are either Chinese or English, we recruited 20 college students who were fluent in both languages to annotate the data. We selected bilingual annotators to reduce bias in stance judgment induced by differences in the language spoken. Before assigning the task, we provided the codebook and tutorial examples to train the annotators and discuss with them. Once they were comfortable with the requirements and the task, we proceeded to formal data annotation. As stance is subjective, to ensures the annotation quality, we conducted 3 rounds of *verification* for Q1, the single option question. Figure 1 shows the annotation and verification processes.

A total of 8000 posts from 4 platforms, with 2000 posts per platform, were labeled in our annotation. In the first round (*R_1_*), we randomly partitioned 20 annotators into 10 groups of 2 annotators each and assigned each group to 800 selected posts (200 from each platform). After annotation, we compared the labeling results within the groups. When 2 annotators labeled a post with the same stance, the task for the post was considered complete. We calculated the inner-annotator agreement between the 2 annotators in the first round using the Cohen κ score.

Instead of requiring 2 annotators to select the same options in Q2, which would be very challenging, we calculated a weighted score for each option based on their answers as follows:



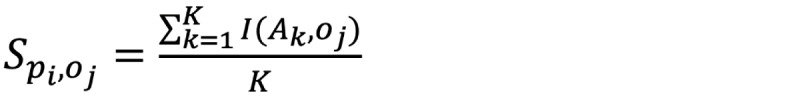




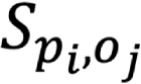
 represents the score of option *o_j_* in post *p_i_*, *i* is the post number from 1 to 800, *j* is the option number from 1 to 7 (see the abovementioned coding questions), *A_k_* represents the set of options that annotator *k* chooses, and *K* is the total number of annotators who labeled post *p_i_*:



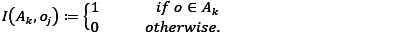



We sent all posts with conflicting stance labels into a second round of annotation (*R_2_*) for tie-breaking purposes. In this round, we selected another 10 annotators as adjudicators. If a post received 3 different stance labels in 2 rounds of annotation, we sent it to the third round of annotation (*R_2_*) for further tie breaking. Multiple rounds of annotation can reduce manual effort and improve efficiency. However, some posts received 2 stance labels in *R_1_*, whereas some other posts received 4 stance labels in *R_3_*. It is possible that the posts that received the same stance labels in *R_1_* could obtain different labels from 2 (different) annotators, which may have made the annotations unreliable. To examine the extent to which such cases existed, we randomly selected 5% of such posts and assigned them to 2 annotators who had yet to label them for a verification process.

For posts with stances settled beyond *R_1_*, we extracted the answers to Q2 from the 2 annotators who determined their final stance label. Once we calculated the weighted score 
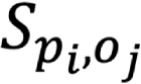
 for each option *o_j_* in each post *p_i_*, from Q2, we estimated 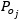, the probability that an issue (corresponding to option *o_j_*) was raised in a web-based platform regarding a particular stance. 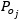 was calculated as follows:



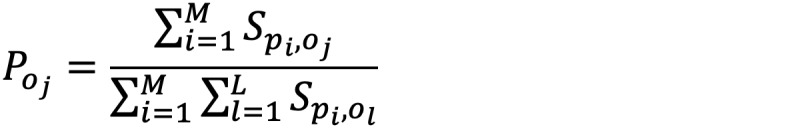



Where *M* represents the total number of labeled posts for a platform, and *L* represents the number of options in Q2.

### Stance Language Analysis

After comparing the stance differences across the 4 platforms, we continued to examine how the language used in the posts differentiated supporting and opposing stances across the platforms. To perform this analysis, we focused on annotated data with only supporting or opposing stances. We removed stop words and extracted the lemma form of each word. We then calculated the saliency [40] *s (w)* for a word *w* in each platform as follows:



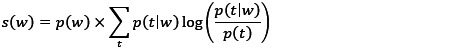



In the abovementioned equation, *t* refers to either a supporting or opposing stance, and *p* represents the probability of *w* within the data corpus. The larger the saliency value, the more informative the word is for differentiating among stances. We retained the 50 most informative words for each platform. For each selected word *w*, we used the relevance score [41] to determine how informative it was to a stance *t*, which is defined as follows:



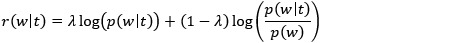



In the abovementioned equation, *λ* is used to control the trade-off between the word frequencies within a stance and the word importance in distinguishing 2 stances. Here, a small *λ* highlights rare but exclusive words for a stance, whereas a large *λ* highlights frequent but not necessarily exclusive words for the stance. In this study, we set *λ*=0.6, as suggested by Sievert and Shirley [41].

## Results

### Data Preparation

Table 2 summarizes the data collected for this study. Within the first round of annotation, the inner-annotator agreement was 0.44 (SD 0.11), which indicated moderate agreement. The median number of posts published by each author was 1 for all platforms, suggesting that most users only had one post or comment in our data set.

**Table 2:** A summary of the data set collected for this study^a^.

Platform and users			Post type		Posts		Posts per user, mean (median)^b^		Post length, mean (SD)^c^
**Sina Weibo**									
	2800	Microblog		4941		1.8 (1)		81.4 (76.8)	
	83,265	Comment		131,126		1.6 (1)		16.6 (17.5)	
**Twitter**									
	24,960	Tweet		47,147		1.9 (1)		14.1 (4.2)	
**Reddit**									
	866	Submission		3205		3.7 (1)		71.0 (324.6)	
	11,678	Comment		22,417		1.9 (1)		43.3 (65.9)	
**YouTube**									
	31,237	Comment		48,172		1.5 (1)		36.1 (60.4)	

^a^On the basis of the properties of each platform, the posting type varies.

^b^Represents the average number (median) of posts per user in each platform.

^c^Represents the average word count (SD) of each post.

### Topic Analysis

#### Topics on Twitter and Reddit

On the basis of our criterion, we set K^*^ to 15 for the topic analysis. We refer the reader to Multimedia Appendix 1, Tables S1-S3, for further details. Table 3 reports on the 15 extracted topics; the most representative words; expected topic proportions (the probability of each topic in all the posts); and the 5 summarized themes, which were summarized by authors (CN and ZY) by manually reviewing the posts with the highest probability, and the difference in topical prevalence between Twitter and Reddit. The most representative words per topic were based on the rank ordering of their probabilities.

**Table 3:** Topics, top words, and the 5 summarized themes.

Theme and topic		Top representation words	ETP^a^	Difference (SD)^b^
**Communication**				
	11	*just*, *realli*, *happen*, *get*, *your*, *like*, *one*, *actual*, *yeah*, and *guy*	0.066	−0.092 (0.001)
	9	*think*, *that*, *thing*, *dont*, *right*, *doesnt*, *cant*, *anyth*, *bad*, and *good*	0.059	−0.090 (0.001)
	5	*can*, *even*, *genet*, *point*, *know*, *well*, *someth*, *understand*, *way*, and *still*	0.058	−0.078 (0.001)
	6	*daddi, [curse word #1], look, cock, [curse word #2], littl, long, talk, perfect,* and *want*	0.038	−0.039 (0.001)
**Discussions**				
	15	*use, diseas, crispr, cell, mutat, technolog, cancer, ccr, techniqu,* and *cure*	0.071	0.013 (0.001)
	1	*gene, edit, hiv, human, embryo, risk, may, twin, genom,* and *studi*	0.064	0.010 (0.001)
	14	*peopl, kid, child, parent, children, dont, rich, alreadi, isnt,* and *wouldnt*	0.059	−0.099 (0.001)
	13	*ethic, now, scientif, experi, said, done, govern, public, need,* and *communiti*	0.049	−0.035 (0.001)
	7	*will, year, futur, chang, come, human, today, –izumi, time,* and *permalinksavecontextful*	0.047	−0.019 (0.001)
	8	*alphian, omegian, planet, civil, ampxb, galaxi, low-level, omega, cosmic,* and *betian*	0.017	−0.016 (<0.001)
**Gene editing**				
	12	*geneedit, genomeedit, crisprca, crispr, tool, geneediting..., genetherapi, amp, genet,* and *genom*	0.121	0.184 (0.002)
**News**				
	3	*gene-edit, scientist, chines, babi, claim, crispr, report, jiankui, scandal,* and *miss*	0.144	0.168 (0.002)
	4	*babi, news, gene-edit, first, moratorium, creat, world, controversi, world,* and *nobelist*	0.089	0.074 (0.002)
	10	*china, crisprbabi, research, scienc, confirm, jiankui, halt, stanford, investig,* and *say*	0.080	0.066 (0.001)
**Web-based posting**				
	2	*comment, pleas, post, question, automat, thank, remov, bot, moder,* and *thought*	0.038	−0.046 (0.001)

^a^ETP: expected topic proportion.

^b^Difference represents the difference in the prevalence of the topic between Twitter and Reddit, where a positive (negative) value suggests a topic was more frequently discussed on Twitter (Reddit). All the differences were significant with *P*<.001 according to a 2-tailed paired *t* test.

Five themes were generated by the authors: *communications* (topics refer to regular web-based chatting), *discussions* (topics related to the discussion of this event), *gene editing* (topics of gene editing technique), *news* (topics of #GeneEditedBabies), and *posting on the web* (topic of common web-based communities’ words). Topics with positive (negative) scores were more often discussed on Twitter (Reddit). For example, it can be seen that topic 12 from the *gene-editing* theme exhibited the highest score, followed by topics 3, 4, and 10, which are all part of the *news* theme. This suggests that Twitter is more dedicated to the sharing of news. By contrast, topic 14 exhibited the lowest negative score, a topic in the *discussion* theme about people and children. Topics 11, 9, and 5 of the *communication* themes also exhibited negative scores, which suggests that they were more likely to be mentioned on Reddit as well. The above findings clearly demonstrate the different topics on the 2 platforms.

#### Topic Temporal Trend Analysis

**Figure 2:**
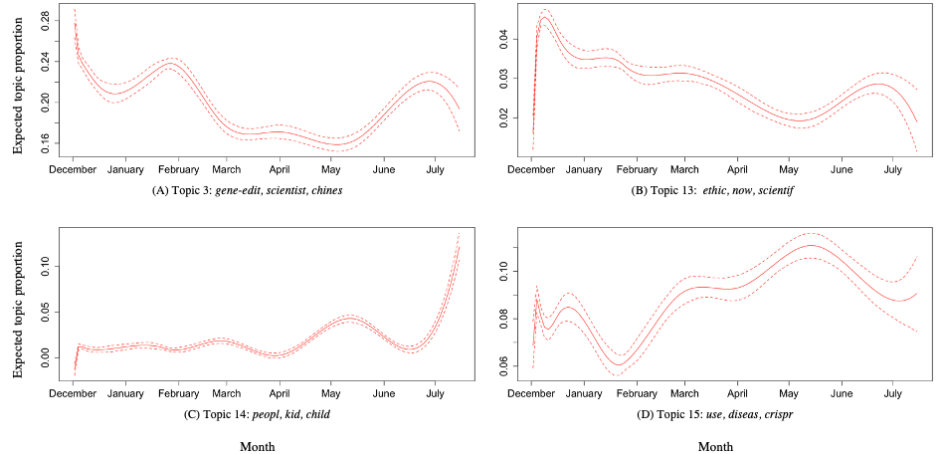
Topic temporal trends for 4 topics generated by applying structural topic modeling on Twitter and Reddit data. The solid line represents the mean expected topic proportion and the dashed lines represent 1 SD. The x-axis corresponds to the posting time, whereas the y-axis corresponds to the expected topic proportion obtained from structural topic modeling. (A) to (D) represent the monthly changes in the expected topic proportion of Topic 3, 13, 14 and 15, respectively.

Figure 2 illustrates the temporal trends for the 4 representative topics derived from Twitter and Reddit. We interpreted the trends by manually examining the posts with the largest probabilities for each topic during a specific period. Note that the scale of the *y*-axis for each topic that we selected in Figure 2 varies because of different expected topic proportions (Table 3).

Topic 3 (in Figure 2) mainly refers to the scientist Jiankui He in this event. After the beginning of the event, the popularity of this topic decreased sharply but then experienced an increase in February 2019. This increase corresponded in time to a rumor that *Chinese gene-editing scientist missing amid rumors of arrest: report says Chinese scientist He Jiankui has been arrested* on Twitter. He again attracted attention in the news in June 2019 because of an announcement by Denis Rebrikov at the Kulakov National Medical Research Center in Moscow, who was planning to repeat his experiment once he obtained official approval [42].

Topic 13 communicates the public’s concerns about the event (eg, legal issues). This topic experienced a decreasing trend as the #GeneEditedBabies event became outdated. However, it experienced a peak around May 2019 because of an article published in the journal Nature: *CRISPR babies: when would the world be ready?* [43], which stirred a discussion on Reddit.

The temporal trend for topic 14, which focuses on human rights and was more likely to be discussed on Reddit, exhibited an increasing trend, with a local peak during May 2020 when a Reddit user shared an article with the title of *What we risk as humans if we allow gene-edited babies: a philosopher’s view* [44], which received >600 comments. The popularity of this topic continued to rise rapidly in July 2020, perhaps because of the news of *Five couples lined up for CRISPR babies to avoid deafness* [45], which received numerous retweets on Twitter.

Topic 15, which focuses on gene-editing technology, grew over time, with a peak around May 2019. The peak of its popularity appeared to coincide with news related to how gene editing helps cure disease. For example, 1 such news story posted on Twitter in May 2019 was *Researcher Brian Liau and his team combine CRISPR gene-editing technology with chemical profiling to tease out acute myeloid leukemia mechanisms* [18].

#### Topics on YouTube and Sina Weibo

We also applied STM to YouTube and Sina Weibo data sets to generate K^*^=15 topics using the same criteria for processing Twitter and Reddit data sets, respectively. The three most popular topics on YouTube were summarized as visual arts (*video*, *gattaca*, and *episod*), thoughts (*want*, *get*, and *think*), and human evolution (*human*, *speci*, and *evolut*). The top three popular topics on Sina Weibo were news (*事情 event, 科研 research,* and *国际 international*), science (*科学 science, 科学家 scientist,* and *成果 result*), and concerns (*法律 law, 胚胎 embryos,* and *人体 human*). Other topics on YouTube included mentions of race and culture (keywords: *tan*, *skin*, *cultur*, *hair*, and *racist*), and general thoughts (*peopl*, *talk*, *feel*, and *understand*). By contrast, Sina Weibo included topics presenting strong negative emotions, such as strong opposition to the event (*出生 born*, *死 death*, *可怜 pity, 毁灭 destroy, 人性 humanity,* and *危害 danger*) and the discussion on inequity between the rich and poor (*穷人* poor, 富人 rich, 普通人 normal people, and *小白鼠 experimental mice* [used as a metaphor]).

### Stance Analysis

#### Annotation Results

**Figure 3:**
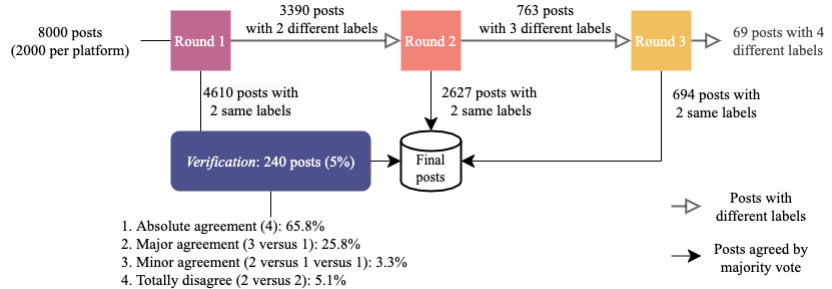
The annotation process for 8000 posts. Note that the 69 posts with 4 different labels were removed from further analysis.

Figure 3 illustrates the labeling process. Both annotators agreed in 57.62% (4610/8000) of the posts that were labeled in the first round. After the second round of annotation, 3 annotators remained in disagreement on 22.51% (763/3390). The 9% (69/763) of posts that were annotated with 4 different options were removed because of the inability to reach any consensus. Regarding verification, 65.8% (158/240) of the examined posts received the same labels as the first round (ie, absolute agreement); 25.8% (62/240) received 1 different label (ie, major agreement); 3.3% (8/240) received 2 different labels (ie, minor agreement); and only 5.1% (12/240) received 2 of the same labels, which differed from the original, suggesting a reliable labeled data set for further analysis.

#### Stance Distribution

We calculated the number and percentage of each stance in each platform among the posts that clearly had a stance in the annotated data. The percentage of removed posts with *no clear stance* was 42.8% (853/1993) on Sina Weibo, 59% (1174/1990) on Twitter, 54.4% (1070/1967) on Reddit, and 45.6% (902/1978) on YouTube. Although all 4 platforms generally contained more opposing stances than other stances, the ratios of those stances were quite different. Specifically, Sina Weibo and Twitter exhibited similar rates of opposition (approximately 84.91% [968/1140]); however, Sina Weibo’s rate of support was almost double that of Twitter (37/816, 4.5%). In contrast, Reddit and YouTube had much lower (higher) rates of opposition (support) at 61.25% (550/898; 257/898, 28.62%) and 52.6% (567/1078; 405/1078, 37.6%), respectively. Sina Weibo exhibited a lower rate of neutral stance (70/1140, 6.1%) than the other 3 platforms (all of which were around 10%). It is notable that, although both Twitter and YouTube have broader international user coverage, YouTube exhibited a more divided stance toward #GeneEditedBabies. We suspect this may be because of the fact that YouTube presents its content in videos, such that users can comment under them and share conflicting ideas in an irrelevant manner. By contrast, Twitter is more akin to a news (re)distribution center, where it is difficult to support a deep discussion.

Although a post-level analysis of stance may be biased by superusers who publish substantially more posts than others, we found that within the annotated posts with a clearly expressed stance, >95% of users had only 1 post (Sina Weibo: 1005/1057, 95.08%; Twitter: 726/755, 96.16%; Reddit: 822/858, 95.8%; and YouTube: 965/1012, 95.35%), and no users had >5 posts. This indicates that the influence of superusers on our findings based on randomly sampled data is limited.

#### Concern Analysis

##### Supporting Stance

**Figure 4:**
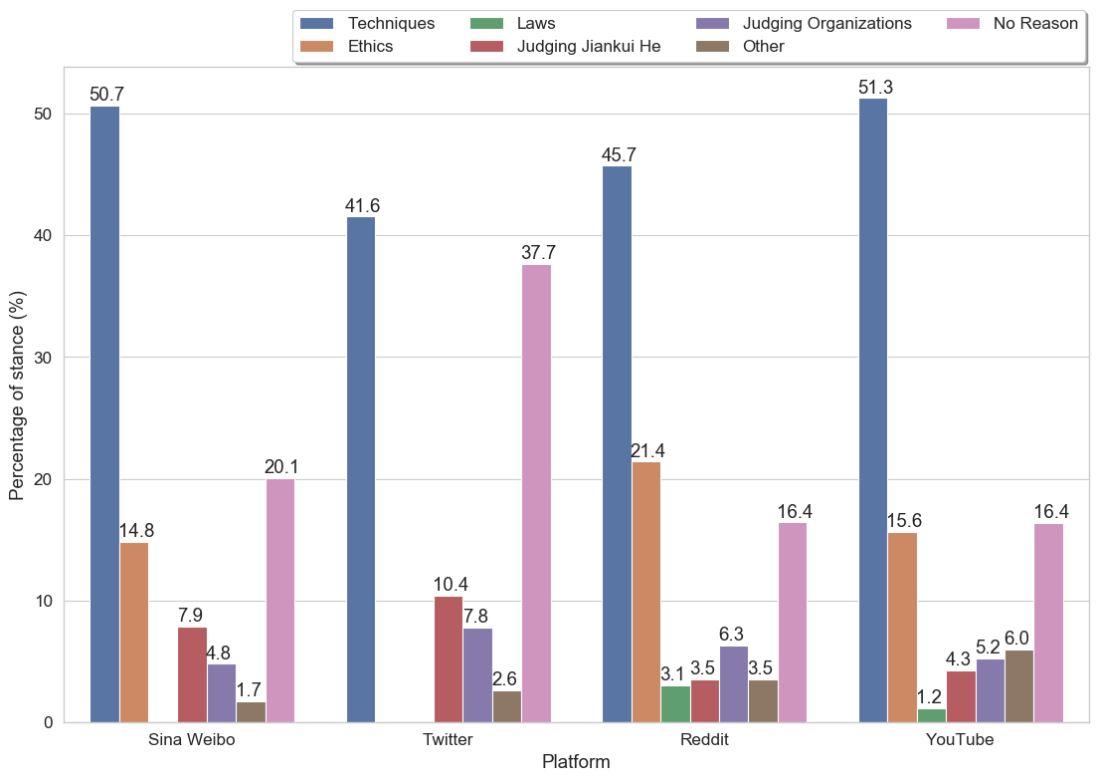
The reasons for supporting stances within 4 platforms.

Figure 4 shows that *techniques* were the primary consideration when posts supported the #GeneEditedBabies event on each web-based platform. The rates of Sina Weibo and YouTube posts supporting this event were slightly >50%, whereas those for Twitter and Reddit were closer to 40%. A qualitative examination of the related posts suggests that users believed the event could help advance scientific knowledge for clinical trials using gene-editing technologies and, thus, create an opportunity to cure severe diseases and benefit humans in the future. A Sina Weibo user commented the following:

解剖学最早也是反人类的， 遭到大多数人的反对，[...]， 恭喜先生走出新的世界纪录。(Anatomy was also anti-human at the earliest and was opposed by most people, [...], congratulations to Mr. for stepping out of the new world record.)

post 1, Sina Weibo

It should be noted that *no reason* was the second most selected option for Q2 on YouTube (76.5/467, 16.4%), Sina Weibo (23/114.5, 20.1%), and Twitter (14.5/38.5, 37.7%) and the third most selected option for Reddit (51/311, 16.4%). The difference in *no reason* ranking within the platforms may be as Reddit discussions are more likely to compose longer posts than the discussions on the other 3 web-based platforms (Table 1) and, thus, have more of a chance to share reasons in their posts. Ethics was another critical consideration when posts supported this event on Reddit (66.5/310.5, 21.4%), YouTube (73/467, 15.6%), and Sina Weibo (17/114.5, 14.8%). The users in these posts did not believe that this event lacked ethical concerns. Instead, these users intimated that the event could help patients with genetic diseases. The two posts indicated the following:

It’s not morality, it’s cowardice to seek permission from 30 governing bodies before taking every step. [...] No risk, no reward.

Post 2, Reddit

Nice work! [...] If you can help parents with a genetic disorder have a healthy baby, I don’t understand why people are so upset.

Post 3, YouTube

We also found posts expressing a supporting stance directly based on the aspects of Jiankui He or organizations (eg, He’s institute or country); posts on Sina Weibo and Twitter tended to talk about Jiankui He himself, whereas Reddit and YouTube tended to say more of his organizations. The following two posts offer examples in these categories:

总有人要付出代价。 如果你不迈出第一步，后面的人就永远不敢前进。(There is always someone who has to pay the price. If you don’t take the first step, the ones behind will never dare to move forward.)

Post 4, Sina Weibo

China will leave us behind because we refuse to edit human genes like this.

Post 5, YouTube

Some Reddit and YouTube posts supported this event as they believed it could help push forward new strategies and regulations for gene therapy, as indicated in post 3.

##### Opposing Stance

**Figure 5:**
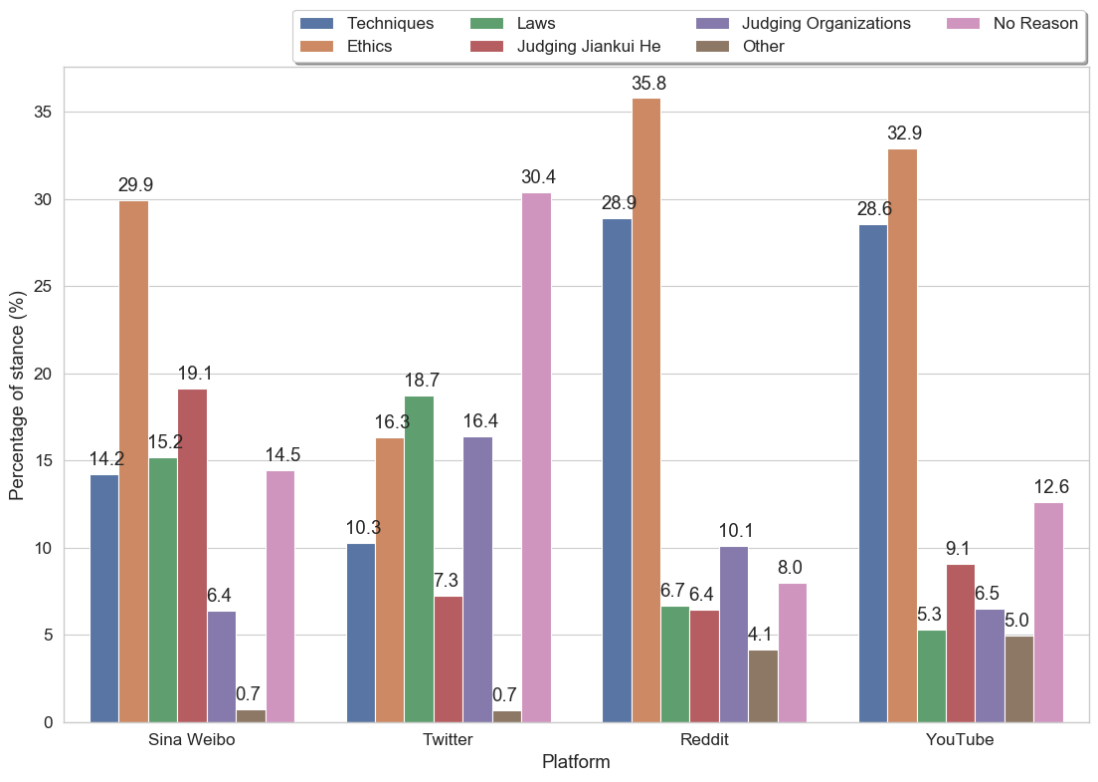
The reasons for opposing stances within 4 platforms.

Figure 5 illustrates the distribution of the issues identified in each platform for the opposing stance. Although there were many comments without any reason, especially on Twitter, ethics was the primary concern for Reddit (259/724, 35.8%), YouTube (235/714, 32.9%), and Sina Weibo (376.5/1258.5, 29.92%). A YouTube opposer said the following:

Who owns the rights to products and technologies? [...] I don’t think it’s been adequately addressed, nor is there transparency in the scientific community about it.

Post 6, YouTube

The fact that the technique is relatively new and could lead to many unknown and serious issues was another consideration in Reddit (209/724, 28.9%), YouTube (204/714, 28.6%), and Sina Weibo (179/1258.5, 14.22%). A Redditor wrote the following:

Genes are complex. So, if it doesn’t work, it’s as if you’re not fighting the infection properly and there’s a higher risk of dying.

post 7, Reddit

However, Sina Weibo had more posts and insults (240.5/1258.5, 19.1%) that were not directed at the event or the technology but were only directed at the individual (Jiankui He). One such quote is as follows:

说白了， 还是利益熏心!! 一身的铜臭味!! (To be straightforward, (He) was too greedy and only cared about the money!)

post 8, Sina Weibo

Notably, Twitter is quite different from the other platforms in that the major considerations mentioned in this platform were laws (151/806.5, 18.7%), organizations (132/806.5, 16.4%), and ethics (131.5/806.5, 16.3%). Techniques (83/806.5, 10.3%) had a smaller percentage than those exhibited in Sina Weibo and much less than on YouTube and Reddit. A possible explanation for this phenomenon is that many users directly tweeted the news without attaching their own commentary, whereas the discussions on the other platforms were more likely to comment under either video or other posts.

#### Language Analysis

**Figure 6:**
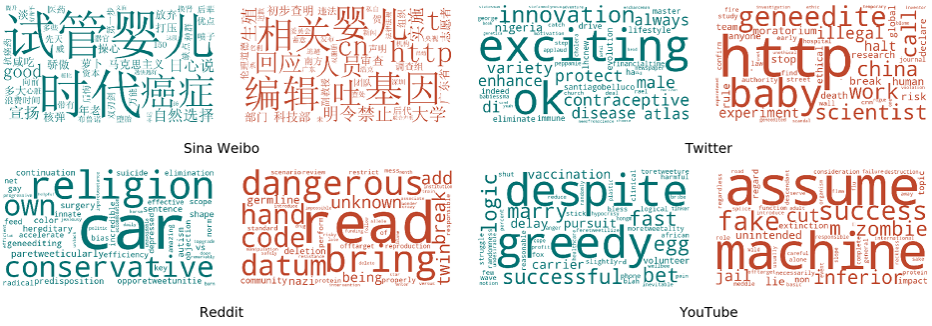
Salient words for supporting (green) and opposing (red) stances within each web-based platform. A larger font size suggests a higher relevance of the associated word in the corresponding stance and platform. Note that all words presented here are in their lemma form.

Figure 6 illustrates the word clouds for each platform, with a larger font size indicating a more informative word.

#### Sina Weibo

The most informative terms used by Sina Weibo supporters were *试管婴儿* (in vitro fertilization)*, 自然选择* (natural selection)*, 癌症* (cancer)*, 时代* (time), good*, 日心说* (heliocentrism)*, 宣扬* (advocate)*, 骄傲* (pride)*, 核弹* (nuclear bomb)*,* and *医药* (drug). These people argued that the event might be part of natural selection, and from an ethical perspective, there might be no difference between in vitro fertilization and gene-edited babies because of human intervention. Some people believed this was good and that society should be proud as they have the potential to cure diseases such as *cancer*. Others argued that gene editing was similar to dual use technologies [46], such as *nuclear* technology, which could be used to create bombs but also be beneficial for energy development; eventually, it would be accepted just as heliocentrism. By contrast, opposers were more likely to mention words related to law or stakeholders, such as *基因编辑* (gene edit)*, 广东省* (Guangdong province)*, 央视* (CCTV)*, 非法* (illegal)*, 明令禁止* (clearly forbidden)*,* and *审查* (governance)*.* It should be noted that 吐 (throw-up) is a common Chinese term in Sina Weibo that expresses an emotion in strong opposition.

#### Twitter

Supporters used many positive words such as *exciting, OK, innovation, protect,* and *enhance*. We found that male contraceptives were also mentioned as they, together with gene editing babies and other findings, were reported as the most exciting innovations of 2018, and the link to the web-based version of this report received many retweets. By contrast, people with an opposing stance used words that were more relevant to this event specifically, such as *baby, geneedite, scientist, china, illegal, death, break, halt, stop, rule,* and *risk*. In this regard, Twitter shared similarities with Sina Weibo in that opposers from both platforms quote news or web-based links (eg, *http, cnn, cnet* on Twitter and *http* and *CCTV* in Sina Weibo).

#### Reddit

Posts on Reddit were more multidimensional and tended to be more thoughtful than posts on other platforms. Supporters discussed this event from the perspective of religion, using terms such as *conservative*, and argued that they did not see any religion-related issues. They also mentioned *hereditary*. They believed that this technology could be used to cure autism or certain predispositions. They also used positive terms such as *effective* and *efficient*. In addition, these supporters likened this technique to the increase in deaths when automobiles were first introduced. The words in the supporting stance also demonstrate that Reddit posts were likely to write deep or scientific, dialectical explanations to express supporting opinions. By contrast, the opposing views included words such as *read, bring, datum, unknown,* and *add*, expressing their concerns about the unknown effects that such a technique might bring to the human gene pool. These opposers also used more technical terms such as *germline, off target, protein, allele, reproduction, deletion,* and *intervention*. Many of them believed it was dangerous, comparable with the strong opposing emotion word *吐 (throw up)* used on Sina Weibo.

#### YouTube

The posts on YouTube invoked a very different language style compared with posts on other platforms. For example, supporters used the term *successful* in expressing their supporting stance and argued that gene-editing techniques will make people healthier and live longer, just as vaccines do today. Although the supporters also used terms that appear to be in opposition, such as *despite, greedy, harmful,* and *struggle*, a closer examination of the related posts showed that these posts presented 2 stances but with a solid preference for a supporting stance. By contrast, opposers on YouTube were more likely to use dramatic words such as *zombie, machine, extinction, and inferior* and conspiracy-related words such as *assume, fake, and lie*. When talking about the law issue, these posts tended to use the word *jail*.

In summary, a comparison of informative terms shows a clear distinction in the language used in these 4 platforms. Moreover, the difference in language occurred in both the supporting and opposing stances toward this event. A possible explanation for this distinction is that it may be an artifact of user populations with different cultures or demographic backgrounds. We believe that this further supports the need to consider the public’s stance from multiple diverse web-based platforms to form a more comprehensive picture. There are certainly other possible explanations, such as the fact that these differences may be because of the political stances or related screening policies of each individual platform. However, further investigation is necessary to determine whether this conjecture is correct.

## Discussion

### Principal Findings

This study introduced a novel, cross-cultural, and cross-regional investigation of the public’s stance toward the #GeneEditedBabies event by using data from 4 popular web-based social media platforms. We found that the platforms focused on different topics and that some had clear temporal trends around the news associated with this event and related techniques. In addition, we found that opposition to this event was high in general; however, the stances varied across platforms. Among the discussions that expressed a clear stance, Twitter exhibited the largest percentage of posts (701/816, 85.9%) in opposition, followed by Sina Weibo (968/1140, 84.91%), Reddit (550/898, 61.2%), and YouTube (567/1078, 52.59%). The main reasons people supported this event were as the techniques were thought to be scientific advances that have the potential to cure debilitating genetic diseases. By contrast, the main reasons for opposition were ethical and legal concerns. The variation we observed may stem from the differences in the cultural and demographic backgrounds of the user base in each platform.

### Comparison With Prior Work

#### Earlier Stance Analysis on Gene Editing

Our findings regarding Sina Weibo and Twitter are aligned with a conclusion in a recent survey of responses to a hypothetical, norm-violating application of gene-editing technology that such applications would *invite public backlash that can spill across national boundaries* [47]. However, as we demonstrated, Reddit and YouTube still had a substantial proportion of posts supporting this event. Other survey-based studies have also found that people tend to have supporting but cautious attitudes toward HGE. For example, 1 study showed that adults from Australia were comfortable with editing human and animal embryos but only for research purposes and to enhance human health [48]. Similarly, Japanese users generally accepted the use of genome editing for disease-related genes; however, many were concerned about its risks [49]. Although many Dutch adults responding to a survey considered the risks of GGE to be substantially greater than its benefits, they may approve of using GGE if it is sufficiently safe and effective and used for a disease instead of enhancement [50]. In addition, another survey based on the responses from 1537 participants across 67 countries (87% of whom were White and mainly from the United States, Australia, Canada, and the United Kingdom) found that respondents generally supported GGE for medical applications, and resistance was mainly reported by people with religious beliefs or working experience in genetics [51].

However, as a recent review of nationally representative public opinion surveys summarized [52], many of these surveys did not capture the views after the #GeneEditedBabies event, *which could have raised awareness of HGE or affected views of edits among public*. In addition, the authors of the review advocated collecting more data from around the world to capture the views of different segments of populations. In this regard, our multiplatform investigation provides more insight into how people with different cultures and from different regions discussed this event, findings that can help fill the gap in this field.

#### Analysis of the Event Using Web-Based Data

Several studies have investigated public discussions using data from social platforms. For example, Calabrese et al [53] examined the perceptions of CRISPR on the web through the application of a semantic network analysis of Twitter messages, and Müller et al [26] analyzed tweets about CRISPR or Cas9 technology and trained machine learning models to classify tweets. However, these studies used data from Twitter only, which was not easily accessible in mainland China. Moreover, they focused on the sentiment of tweets (eg, positive or negative) and the type of subject (eg, applying the technology to humans as opposed to other organisms) instead of the stance (eg, in support or in opposition) and greater understanding of concerns (eg, why hold this stance?). Furthermore, the #GeneEditedBabies event was not the main focus. Zhang et al [27] compared the differences in language used to discuss gene editing before and after this event on Sina Weibo. Liu and Lapata [54] conducted a sentiment analysis of news reports and tweets about this event that were collected from Google, Baidu, Sina Weibo, and Twitter about this event. However, all of these investigations are fundamentally different from ours in that they selected various platforms for data collection and focused solely on sentiment analysis, which is insufficient to characterize the stance expressed in a comment.

#### Web-Based Multiplatform Data Analysis

Multiplatform studies are useful for reducing the bias caused by a single platform or a small amount of data. In particular, multiplatform data resources can enrich analysis and optimize the training results of classifiers [55,56]. For example, Schifanella et al [57] leveraged the contextual information carried by visuals to decode the sarcastic tone of multimodal posts by using images and texts from Instagram, Tumblr, and Twitter. In addition, most multiplatform data analyses targeted a particular topic, such as travel (eg, discussion about hospitality and tourism [58]), business (eg, the preferences and discussions of e-cigarette users [59]), and health (eg, a study of symptoms of depression and anxiety of American young adults [60]). Our study contributes to this area of research by using multiplatform data to conduct a reasonable and comprehensive analysis of stances across various cultures, regions, and network environments.

### Limitations and Future Work

However, there are certain limitations in our study that can serve as the basis for future research. First, although we focused on 4 popular publicly accessible platforms, there are other major web-based platforms (eg, Facebook), the data of which could be used to enrich the analysis. Second, the set of keywords we relied upon was limited to the names of the event and the scientist. Although we believe that these are likely to cover most of the perspectives that can be detected, we did not conduct a pretest to confirm if this was the case. Third, we calculated the percentage of stances based on randomly selected posts and showed that superusers had a limited impact on our findings. Future work may consider directly sampling all users to avoid this issue. Fourth, although our annotated data set is sufficiently large to obtain insight into the public’s stance, it would be interesting to conduct stance temporal trend analysis with more labeled data. Finally, our findings reflect only the opinions of people who use web-based social media platforms to express their stances on this event. It is necessary to investigate how their perspectives relate to people who do not post on social media.

This project laid the foundation for a wide array of future studies. First, to analyze the language used, we applied a saliency word cloud to show the differences in language among posts from different stances and platforms. Alternative methods, such as semantic networks [61], could be considered in future work to investigate a different perspective of topic modeling; for example, the relationships among the words. Second, it would be useful to determine whether the public’s stance on gene editing changed as a result of this event. A way by which this could be accomplished would be to compare posts about gene-editing technology before and after the #GeneEditedBabies event across social media platforms. Third, although we focused only on post-level content for analysis, it will be useful to attach conversations, such as the comments under the posts in each platform, to enrich this research.

### Implications and Conclusions

This study has several notable implications. First, this study shows that web-based social media platforms can serve as efficient tools for tracking people’s reactions to a series of news regarding a public event in a timely manner. However, such reactions can vary depending on the platform from which the posts come. Using multiplatform data to study public events can help to obtain a more comprehensive understanding of people’s stances worldwide. Second, web-based posts exhibit more divided stances on, understandings of, and interpretations regarding a cutting edge but controversial technique than those expressed by academic professionals. This adds further weight to the need to listen to public voices and increase public engagement in policy formation beyond the scientific community [6]. Finally, the public’s observed stances and the factors that web-based posts considered can both help guide the development of promotional materials to improve awareness and understanding of this technique for posts on different platforms [62] as well as take public concerns more fully into account.

Although we focused on studying the public’s stance regarding this specific event, our annotation methods and analysis strategies can be readily adapted to investigate other social events (eg, Black Lives Matter or presidential elections) and public health promotions (eg, cancer screening or COVID-19 vaccination) in a cross-cultural or cross-regional manner.

## Appendix

Multimedia Appendix 1 The top words of experiments of evaluating proper K for structural topic modeling and task examples of questions annotators got before formally starting annotation.

## Abbreviations

API: application programming interface
Cas9: CRISPR-associated protein 9
CRISPR: clustered regularly interspaced palindromic repeats
GGE: germline gene editing
HGE: human gene editing
STM: structural topic modeling
