# Supplementary material for: The Public Perception of the #GeneEditedBabies Event Across Multiple Social Media Platforms: Observational Study
Source: J Med Internet Res. 2022 Mar 11;24(3):e31687. doi: 10.2196/31687 (PMC8957000; doi:10.2196/31687)
Supplement: Multimedia Appendix 1 [file jmir_v24i3e31687_app1.docx]

# Supplemental Material for

# “Beyond the Academic Opinion on #GeneEditedBabies: How the Public Perception of a Controversial Scientific Event Differs Across Web-Based Social Platforms”

Congning Ni^1^, ME; Zhiyu Wan^1,2^, PhD; Chao Yan^1^, MS; Yongtai Liu^1^, MS; Ellen W. Clayton^3,4,5^, MD, JD; Bradley A. Malin^1,2,3,6^, PhD; Zhijun Yin^1,2,3^, PhD

^1^Department of Electrical Engineering and Computer Science, Vanderbilt University, Nashville, TN, United States

^2^Department of Biomedical Informatics, Vanderbilt University Medical Center, Nashville, TN, United States

^3^Center for Genetic Privacy & Identity in Community Settings, Vanderbilt University Medical Center, Nashville, TN, United States

^4^Center for Biomedical Ethics and Society, Vanderbilt University Medical Center, Nashville, TN, United States

^5^Department of Pediatrics, Vanderbilt University Medical Center, Nashville, TN, United States

^6^Department of Biostatistics, Vanderbilt University Medical Center, Nashville, TN, United States

Corresponding Author:

Zhijun Yin, PhD

2525 West End Avenue, Suite 1412A,

Department of Biomedical Informatics

Nashville, TN, 37203

Email: zhijun.yin@vanderbilt.edu

For each topic number candidate, we calculated the proportions of topics that have semantic coherence and exclusivity scores falling within one standard deviation of mean metric values of semantic coherences ($\geq$ $SC_{avg-sd}=-123.20$) and exclusivities (${\geq E}_{avg-sd}=9.56$). Note that $SC_{avg-sd}$ and $E_{avg-sd}$ are calculated upon all the topic number candidates ranging from 5 to 25. Table S1 summarizes the top three candidates, 21, 15, and 17, based on this criterion.

Table S1. The top three topic number candidates selected after balancing semantic coherence and exclusivity scores.

| K | Number of Topics Within the Defined Region | Percentage |
| --- | --- | --- |
| 21 | 14 | 0.67 |
| 15 | 9 | 0.60 |
| 17 | 10 | 0.59 |

To determine the best model from these three candidates, we relied on the interpretability. Specifically, we presented all the topics and corresponding top words generated respectively, and manually examined each topic. We found that K=21 generated more topics but some of them apparently overlapped with each other (e.g., #Topics 6, 10 and 19 were all talking about CRISPR techniques, See Table S2). We also found that the difference between K=17 and K=15 are not quite obvious, and they share a majority of common topics. For simplicity, we chose K=15 to conduct our further analysis. We provide all the topics for K=17 in Table S3 for readers’ reference.

Table S2. The top 15 words of each topic in model with K=21.

| Topic | Top words |
| --- | --- |
| 1 | book, read, time, interest, great, listen, movi, articl, stori, back, sound, man, seen, like, histori, |
| 2 | hiv, diseas, mutat, cell, risk, ccr, effect, resist, immun, infect, virus, cure, prevent, cancer, target, |
| 3 | year, advanc, ‚Äìizumi, izumi, editdelet, permalinksavecontextful, power, ago, kind, human, earth, comment, next, societi, today, |
| 4 | alphian, omegian, planet, civil, ampxb, galaxi, low-level, omega, cosmic, golden, develop, betian, kappa, high-level, gamma, |
| 5 | issu, children, ethic, super, reason, mani, much, eugen, exact, pretti, potenti, yet, social, women, men, |
| 6 | gene, edit, human, crispr, dna, embryo, genom, tool, use, modifi, germlin, sequenc, valley, silicon, make, |
| 7 | point, chang, might, futur, now, definit, brain, hope, real, god, can, play, find, learn, wonder, |
| 8 | new, experi, ethic, govern, regul, tech, compani, alarm, inventor, debat, oversight, technology\’, babies\’, ban, evad, |
| 9 | crispr, research, controversi, patient, halt, trial, scienc, underway, harvard, work, treat, univers, health, team, week, |
| 10 | crisprbabi, gene-edit, news, via, rogu, death, may, penalti, latest, crispr, stop, panel, face, ban, uproar, |
| 11 | get, well, see, happen, yeah, probabl, one, everyon, everi, mayb, work, tri, yes, shit, els, |
| 12 | peopl, kid, genet, child, parent, rich, alreadi, life, poor, person, differ, choos, afford, moral, popul, |
| 13 | china, jiankui, claim, report, miss, babi, confirm, say, hejiankui, announc, investig, doctor, fame, illeg, whereabout, |
| 14 | technolog, use, techniqu, twin, scientif, said, alter, two, success, hes, paper, open, medic, born, clinic, |
| 15 | will, can, way, live, come, abl, engin, suffer, countri, bodi, money, matter, put, far, access, |
| 16 | gene-edit, scientist, babi, chines, moratorium, world\’, first, scandal, nobelist, creat, mentor, stanford, told, world, call, |
| 17 | pleas, post, russian, nobel, comment, question, biologist, crispr-edit, plan, automat, knew, thank, bot, laureat, moder, |
| 18 | think, dont, thing, just, that, someth, your, know, even, like, doesnt, actual, anyth, realli, problem, |
| 19 | geneedit, genomeedit, crisprca, crispr, tool, genetherapi, geneediting‚ newspaper, amp, genet, genom, repurpos, engin, plant, develop, vertex, |
| 20 | daddi, fuck, cock, fucktoy, look, littl, perfect, talk, want, becom, deaf, least, design, long, age, |
| 21 | isnt, done, sure, take, without, don\’t, guy, meme, understand, generat, idea, test, never, line, modif, |

Table S3. The top 15 words for each topic in model with K=17.

| Topic | Top words |
| --- | --- |
| 1 | will, human, rich, way, societi, intellig, come, speci, countri, kind, popul, world, evolut, superior, advanc, |
| 2 | gene-edit, chines, scientist, babi, china, jiankui, claim, miss, first, world\’, report, world, confirm, say, creat, |
| 3 | alphian, omegian, planet, ampxb, civil, galaxi, low-level, omega, cosmic, golden, betian, high-level, kappa, earth, gamma, |
| 4 | just, well, get, that, right, sure, happen, probabl, yeah, alreadi, need, super, take, everyon, start, |
| 5 | gene, edit, make, don\’t, possibl, said, \’re, cure, made, tri, sound, put, might, find, \’ve, |
| 6 | pleas, post, comment, question, automat, articl, thank, remov, bot, moder, action, opinion, concern, thought, subreddit, |
| 7 | crisprbabi, news, scienc, stori, amp, china\’, new, week, biotech, latest, crispr-babi, book, stop, geneeditsummit, embryo, |
| 8 | gene-edit, babi, scientist, nobelist, scandal, mentor, chines, crispr-edit, stanford, nobel, told, russian, laureat, rule, biologist, |
| 9 | geneedit, genomeedit, crisprca, moratorium, edit, geneediting‚ newspaper genetherapi, genet, genom, amp, plant, engin, gene, crispr-ca, global, |
| 10 | peopl, child, parent, think, kid, your, children, dont, life, deaf, someon, want, can, person, suffer, |
| 11 | crispr, gene-edit, tool, use, patient, trial, human, treat, underway, techniqu, repurpos, first, develop, inventor, cancer, |
| 12 | even, genet, dont, someth, problem, isnt, anyth, bad, understand, argument, wouldnt, poor, know, there, abl, |
| 13 | thing, point, realli, one, lot, though, done, think, idea, mani, didnt, know, alway, imagin, either, |
| 14 | hiv, diseas, cell, mutat, ccr, risk, effect, dna, immun, resist, infect, virus, target, alter, prevent, |
| 15 | year, like, time, ago, see, ‚Äìizumi, izumi, editdelet, permalinksavecontextful, end, shit, month, much, got, comput, |
| 16 | research, experi, controversi, technolog, ethic, scientif, harvard, twin, sperm, help, may, anim, organ, begin, brain, |
| 17 | daddi, fuck, cock, fucktoy, want, give, perfect, littl, long, talk, born, design, becom, age, open, |

We trained the annotators by providing a detailed instructions as well as some samples to help them understand the questions. The task examples are presented below in S4.

Example S4: Task examples of questions annotators got before formally starting annotation.

**Platform**: YouTube

**Post To be Annotated**:

*“Roguish, irresponsible and no respect of life and science. The research has badly violated academic integrity. I am wondering if you are aware about you maybe prosecuted for this selfish action and if you dare to face the justice. This is a total of illegal medical practice!!!”*

**Q1: What is this post’s stance regarding this event? Please select one answer only.**

For Q1, you need to first decide whether the post itself is clearly expressing stances towards this event. Some of the posts may simply talk about topics that are not relevant to the event or they do not clearly show their stances.

| ⮚ Irrelevant or unclear  *(There are no clues in the post that imply the poster’s opinion about the event or Jiankui He)* |
| --- |
| ⮚ Support |
|  |
| ⮚ Oppose |
|  |
| ⮚ Neutral  *(The post must provide some information that suggests that the poster is neutral towards the subject – if the post is neither favorable nor against the subject, then there is no sufficient reason for choosing this answer)* |

Tips:

A. If the post is indeed talking about the gene-edited-babies event, you will then decide whether its stance is “Support”, “Oppose” or “Neutral”. In this example, the post is communicating an opposition stance to this event, so you should choose “Oppose”.

**Q2: In what aspects does the post concern when it expresses its stance? Please select one or more options. (Multi-choice question)**

(There may be more than one reason for the stance, please check all the options that you think reasonable. If some reasons are not covered, choose OTHERS and write the reason with your own words.)

🗶  Ethics

🗶  Laws

🞎  Techniques

🞎  Judging Jiankui He

🞎  Judgements Organizations

🞎  No Reason

🞎  Others __________________

Here are some examples that may include in each option. The examples are not fully covered all the possibilities.

| Option | Post Samples |
| --- | --- |
| Ethics | •  That's not really worth worrying about. Having genetic advantages massively outweighs tiny playground mocking.  •  One comment saying “who cares about ethics it is western culture” The lack of care and caution of this scientist is outrages and he should be jailed |
| Laws | •  in most countries the practise is banned, meanwhile China is a "grey area", hence why he was able do it  •  This is an illegal underground experiment~! |
| Techniques | •  If they can fix or revert hair loss.... i'm in. Ah, and the diseases as well, sure, of course! Off course.... but the first one first. :P  •  I believe there must be others. Maybe CCR5 was the first one worked out. |
| Judging Jiankui He | •  Thanks for taking this step! We are excited to see your progress!  •  贺建奎渣(He Jiankui sucks!)  •  长得就令人不信服[喵喵](Firstly, his face shows he cannot be trusted.) |
| Judging Organizations | •  Chinese don't follow the world ethics anyway.  •  为什么国外做基因编辑可以国内的不行? (why other countries can do gene-editing, but we cannot?) |
| No Reason | •  This is horrible!  •  I support this! |

Tip:

A. Please find the reason why this post supports/against this event. In the example, the author talked about “Roguish, irresponsible and no respect of life and science”, which is about an Ethics concern, and “illegal medical practice”, which is a legal issue.
